# Deletion of the *TNFAIP3/A20 *gene detected by FICTION analysis in classical Hodgkin lymphoma

**DOI:** 10.1186/1471-2407-12-457

**Published:** 2012-10-05

**Authors:** Junko Nomoto, Nobuhiro Hiramoto, Motohiro Kato, Masashi Sanada, Akiko Miyagi Maeshima, Hirokazu Taniguchi, Fumie Hosoda, Yoshitaka Asakura, Wataru Munakata, Naohiro Sekiguchi, Dai Maruyama, Takashi Watanabe, Hitoshi Nakagama, Kengo Takeuchi, Kensei Tobinai, Seishi Ogawa, Yukio Kobayashi

**Affiliations:** 1 Hematology Division, National Cancer Center Hospital, Tokyo, Japan; 2 Section of Microbiology and Immunology, Tokyo Medical and Dental University Graduate School of Health Care Sciences, Tokyo, Japan; 3 Cancer Genomics, Faculty of Medicine, The University of Tokyo, Tokyo, Japan; 4 Pathology Division, National Cancer Center Hospital, Tokyo, Japan; 5 Cancer Genomics Division, National Cancer Center Research Institute, Tokyo, Japan; 6 Early Carcinogenetic Division, Research Institute, National Cancer Center Hospital, Tokyo, Japan; 7 Pathology Project for Molecular Targets, The Cancer Institute, Japanese Foundation for Cancer Research, Tokyo, Japan

**Keywords:** FICTION analysis, Hodgkin lymphoma, *TNFAIP3* gene, Homozygous deletion

## Abstract

**Background:**

The *TNFAIP3* gene, which encodes a ubiquitin-modifying enzyme (A20) involved in the negative regulation of NF-κB signaling, is frequently inactivated by gene deletions/mutations in a variety of B-cell malignancies. However, the detection of this in primary Hodgkin lymphoma (HL) specimens is hampered by the scarcity of Hodgkin Reed-Sternberg (HR-S) cells even after enrichment by micro-dissection.

**Methods:**

We used anti-CD30 immunofluorescence with fluorescence in-situ hybridization (FISH) to evaluate the relative number of TNFAIP3/CEP6 double-positive signals in CD30-positive cells.

**Results:**

From a total of 47 primary classical Hodgkin lymphoma (cHL) specimens, 44 were evaluable. We found that the relative numbers of TNFAIP3/CD30 cells were distributed among three groups, corresponding to those having homozygous (11%), heterozygous (32%), and no (57%) deletions in *TNFAIP3*. This shows that *TNFAIP3* deletions could be sensitively detected using our chosen methods.

**Conclusions:**

Comparing the results with mutation analysis, *TNFAIP3* inactivation was shown to have escaped detection in many samples with homozygous deletions. This suggests that *TNFAIP3* inactivation in primary cHL specimens might be more frequent than previously reported.

## Background

The *TNFAIP3/A20* gene encodes a ubiquitin-modifying enzyme involved in the termination of NF-κB responses, so is a negative regulator of NF-κB signaling [1,2]. *TNFAIP3* is located on chromosome 6q23, and deletion of one allele has been detected in Hodgkin lymphoma (HL) and other B-cell malignancies [3-9].

We previously showed that *TNFAIP3* is a common genetic target in B-cell lymphomas, following an analysis of 265 samples obtained from various B-cell lymphomas using either comparative genomic hybridization (CGH) or *TNFAIP3* mutation analysis. We observed *TNFAIP3* mutations and/or deletions in 31 cases [3]. This previous work also included samples from 24 primary classic HL (cHL) cases, and we performed mutation analysis of micro-dissected CD30-positive Hodgkin Reed-Sternberg (HR-S) cells. This revealed one intronic and four missense mutations, indicating the existence of cHL heterogeneity. However, it is conceivable that we did not detect homozygous deletions, which have been shown in other B-cell lymphoma subtypes and HL cell lines, and that we underestimated the frequency of involvement of *TNFAIP3* in primary cHL cases.

In order to accurately evaluate the frequency of involvement of *TNFAIP3* in cHL, we performed Fzluorescence Immunophenotyping and interphase Cytogenetics as a Tool for the Investigation Of Neoplasm (FICTION) [10] to examine *TNFAIP3* deletions in cHL. A total of 47 cHL cases were examined for the presence or absence of *TNFAIP3* deletions, including 22 of 24 cases with sequence data previously reported.

## Methods

### Samples

The study consisted of 47 cHL biopsy specimens, including 30 from our previous cohort archived in the National Cancer Center (NCC) Hospital between 1997 and 2007, and 17 obtained from The Cancer Institute Hospital of the Japanese Foundation For Cancer Research. This research was carried out in compliance with the Helsinki Declaration, and was approved by the Institutional Review Board at the NCC (20–010).

Twenty-two of the 47 cases had been examined previously by sequence analysis, and four found to have missense mutations. In addition, we examined the sequence of six cases in the present study. Specimens were fixed in formalin or methanol and embedded in paraffin, then cut into thin sections and laid on a glass slide. One section of each specimen was subjected to FICTION analysis and the other to sequence analysis.

### FICTION analysis

Approximately 4-μm-thick sections were immunostained with an anti-CD30 antibody to identify HR-S cells. The CD30 antibody (BerH2) (Dako, Glostrup, Denmark) was diluted 100-fold and incubated overnight at 4°C. The fluorescence labeled antibodies Alexafluor 647 Rabbit Anti-mouse IgG, Alexafluor 647 Goat Anti-rabbit IgG and Alexafluor 647 Donkey Anti-goat IgG, were used as the secondary, tertiary, and quaternary antibodies, respectively (Molecular Probes, Life Technologies Corporation, Foster City, CA). Each of these antibodies was diluted 1000-fold and incubated for 30 min at room temperature (RT).

A BAC clone library was screened to identify a clone suitable for the fluorescence in-situ hybridization (FISH) analysis of *TNFAIP3*. Clone RP11-783B20 (*TNFAIP3* locus, 6q23) was selected based on the best signal/noise ratio upon hybridization to the normal karyotype (Abbot Laboratories, Abbot, IL). RP11-783B20 was labeled with spectrum orange by nick translation (Abbot Laboratories) according to the manufacturer’s instructions. The CEP6 Spectrum Green Probe (Abbot Laboratories) was used to detect the centromere of chromosome 6 (6p11.1-q11) as a reference. Double-color FISH was performed using the Histology FISH Accessory kit (Dako). The hybridization mixture consisted of 2 μl of the *TNFAIP3* probe, 2 μl of the 1/20-diluted CEP 6 probe, 1 μl of Cot1-DNA, and 5 μl of 20% dextran sulfate/4 × SSC. After denaturation at 76°C for 6 min, the solutions were laid onto a glass slide and incubated overnight at 37°C for hybridization according to the manufacturer’s instructions. Nuclear staining was performed with DAPI. We visualized the sections under a four-color fluorescence microscope, BIOREVO (Keyence Corporation, Osaka, Japan).

### Statistical analysis

As the diameter of HR-S cells is several-fold greater than the 4 μm thickness of the FISH sections, the TNFAIP3/CEP6 signal ratio was calculated to evaluate the *TNFAIP3* status in CD30-positive cells. The signal ratio was also calculated in the surrounding normal CD30-negative cells, which were used as a control. We counted the signal ratio for 30 CD30-positive cells and 50 CD30-negative cells. Only CD30-positive cells of large morphology were regarded as HL cells.

The cutoff level to estimate gene deletion(s) was determined by calculating the ratio of the TNFAIP3/CEP6 signal in the subject cells to that in the normal control cells in samples showing obvious deletions from sequence analysis. *P*-values < 0.05 were considered statistically significant.

## Results

In total, specimens from 47 patients were analyzed (Table 1). The predominant type of cHL was nodular sclerosis (NS, n = 28), followed by mixed cellularity (n = 14), lymphocyte-depletion (LD, n = 2), and lymphocyte-rich (LR, n = 3). Of the 47 patients, 19 were male and 28 were female. Disease stages I, II, III and IV were evident in eight, 24, 10 and five cases, respectively. B symptoms were present in 12 cases and absent in the remaining 35 cases. In all, 15 cases tested positive for EBV and the remaining 32 showed a negative result.

**Table 1:** Patient characteristics

**Case #**	**Diagnosis**	**Age (years)**	**Gender**	**Stage**	**B symptom**	**EBER-1**
1	NS	28	F	II	-	-
2	NS	44	F	II	-	+
3	NS	30	M	I	-	-
4	NS	27	F	II	-	-
5	NS	18	F	II	-	-
6	NS	27	M	II	+	-
7	NS	22	M	IV	-	-
8	NS	57	M	III	-	-
9	NS	48	M	III		-
10	MC	29	M	IV	-	-
11	NS	34	M	II	-	-
12	NS	22	F	II	-	-
13	NS	14	F	I	-	-
14	MC	53	M	I	-	-
15	MC	59	M	I	-	+
16	MC	42	M	III	+	-
17	MC	50	M	II	-	-
18	MC	29	F	II	+	+
19	MC	50	M	I	-	+
20	NS	39	F	IV	-	-
21	NS	22	F	II	+	-
22	MC	55	F	I	-	+
23	LD	59	F	I	-	-
24	NS	32	F	II	+	-
25	NS	16	M	III	-	-
26	NS	24	F	III	-	-
27	NS	20	M	II	-	-
28	MC	67	F	IV	+	+
29	NS	26	F	II	-	-
30	MC	73	F	IV	-	+
31	MC	74	F	II	-	-
32	LR	81	F	II	-	-
33	MC	75	F	III	+	+
34	MC	33	F	III	+	-
35	MC	62	F	III	-	+
36	MC	53	M	II	-	+
37	LR	72	M	II	-	+
38	NS	16	F	II	-	-
39	NS	36	F	II	+	-
40	NS	16	F	II	-	-
41	LR	48	M	III	+	+
42	LD	67	M	I	-	+
43	NS	27	F	II	-	-
44	NS	43	F	II	-	-
45	NS	64	M	II	+	-
46	NS	66	F	III	-	+
47	NS	24	F	II	-	+

*NS*, Nodular sclerosis cHL; *MC*, Mixed cellularity cHL; *LD*, Lymphocyte-depleted cHL; *LR*, lymphocyte-rich cHL.

**Figure 1:**
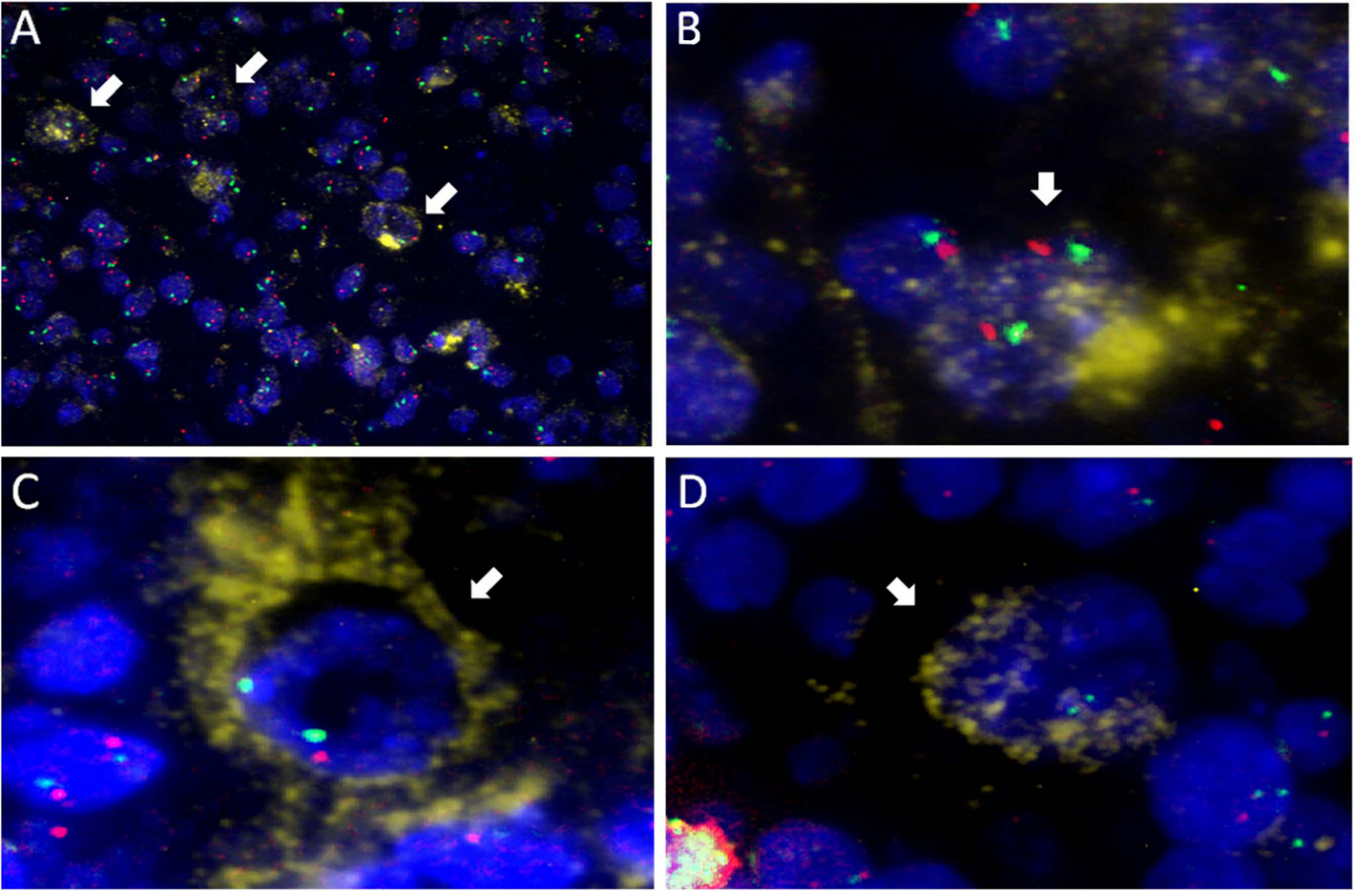
**Results of FICTION analysis. FICTION assay combining CD30-expressing cells (yellow) and FISH probes for A20 (red) and chromosome 6 centromere (green). **White arrows indicate CD30-positive cells. (**A**) Low-power field (×300). (**B**) Normal cells. (**C**) Heterozygous deletion. (**D**) Homozygous deletion (×1,000).

The signals were successfully detected in all cases (Figure 1A-D). The mean numbers of *TNFAIP3* signals and CEP6 signals per CD30-negative cell were almost the same in most cases, except for cases #40, 42 and 45, where the number of *TNFAIP3* signals was markedly lower than that of CEP6 signals, with ratios of 0.86, 0.89 and 0.84, respectively. These cases were excluded from further analysis as the number of available slides was limited and the analysis could not be repeated more than twice. In all remaining cases, the TNFAIP3/CEP6 signal ratio in CD30-negative cells was approximately 1.0.

The mean number of CEP6 signals in CD30-positive cells was 1.2 ± 0.3, which was less than that of CD30-negative cells (1.5 ± 0.1). In addition, the mean number of *TNFAIP3* signals per CD30-positive cell was less than that of CEP6; the mean number of *TNFAIP3*: CEP6 signals per CD30-positive cell was 0.60, significantly lower than that in control CD30-negative cells (*p* < 0.001). The relative numbers of signals determined by FICTION analysis in the 47 cases are shown in Table 2.

**Table 2:** FICTION analysis

**Case #**	**Mutation**	**Control CD30-)**	**HR-S (CD30+)**	**HR-S/ Control**	Predicted allele*
1	W	1.02	0.90	0.88	2
2	W	0.98	0.63	0.64	2
3	1487C>A (T474N)	0.99	0.71	0.72	2
4	W	1.11	0.80	0.72	2
5	W	1.11	0.54	0.49	1
6	W	1.06	0.14	0.13	0
7	W	0.94	0.65	0.69	2
8	W	0.96	0.45	0.47	1
9	W	1.01	0.64	0.63	2
10	W	0.97	0.77	0.79	2
11	W	1.02	0.81	0.79	2
12	ND	1.03	0.22	0.21	0
13	ND	0.99	0.93	0.94	2
14	ND	1.12	0.78	0.70	2
15	ND	1.04	0.83	0.80	2
16	ND	1.05	0.42	0.40	1
17	ND	1.19	1.07	0.90	2
18	ND	1.00	0.67	0.67	2
19	ND	1.17	0.50	0.43	1
20	ND	0.99	0.90	0.91	2
21	ND	0.95	0.21	0.22	0
22	ND	0.97	0.68	0.70	2
23	ND	0.93	0.71	0.76	2
24	ND	0.99	0.70	0.71	2
25	ND	1.00	0.82	0.82	2
26	ND	1.00	0.14	0.14	0
27	ND	1.02	0.32	0.31	1
28	ND	0.99	0.50	0.51	1
29	ND	0.99	0.41	0.41	1
30	ND	0.97	0.76	0.78	2
31	W	1.03	0.45	0.44	1
32	W	1.01	0.88	0.86	2
33	W	0.97	0.52	0.53	1
34	W	0.99	0.52	0.52	1
35	W	1.00	0.93	0.93	2
36	W	0.90	0.63	0.71	2
37	W	0.98	0.43	0.44	1
38	1777G>A (V571I)	1.04	0.72	0.69	2
39	W	1.06	1.00	0.94	2
40	1156A>G (R364G)	0.86	0.15	0.17	ND
41	W	0.91	0.19	0.21	0
42	W	0.89	0.31	0.35	ND
43	W	1.00	0.36	0.36	1
44	W	1.01	0.52	0.51	1
45	W	0.84	0.68	0.81	ND
46	569G>A (STOP)	0.99	0.69	0.70	2
47	W	1.00	0.45	0.45	1
	3/25 (12%)	Mean 1.01	Mean 0.61	Mean 0.60	

* 0: homozygous, 5 cases; 1: heterozygous, 14 cases; 2: non deleted, 25 cases.

W, wild type; ND, not determined.

Cases #1-5 and #31-47 had been sequenced and reported elsewhere. Cases #6-11 were sequenced in this study. Cases #12-30 were not sequenced because of limited or poor quality DNA.

The TNFAIP3/CEP6 signal ratio in CD30-positive cells was divided by that in CD30-negative cells to give the relative TNFAIP3/CEP6 signal ratio; further analysis was then performed to determine whether *TNFAIP3* deletions existed in CD30-positive cells. The relative TNFAIP3/CEP6 signal ratio was distributed into three peaks. Construction of a histogram based on 0.05 increments revealed no cases with relative ratios of 0.25–0.30 or those of 0.55–0.60, which separated the three distribution peaks. We contended that each of these peaks corresponded to the *TNFAIP3* deletion status. The first peak, corresponding to the lowest relative ratios, was considered to represent cases with homozygous deletions; the second peak, corresponding to intermediate ratios, represented cases with heterozygous deletions; and the third peak, corresponding to the highest relative ratios, represented cases with no deletions. Two cutoff levels were thus determined (0.25 and 0.55), distinguishing between cases with homozygous deletions and heterozygous deletions, and between cases with heterozygous and no deletions, respectively.

Using these two cutoff levels, we determined that five of the 44 cases had homozygous deletions and 14 cases had heterozygous deletions. The relative TNFAIP3/CEP6 signal ratios in the former cases (#6, 12, 21, 26, and 41) were 0.13, 0.21, 0.22, 0.14, and 0.21, respectively, suggestive of almost complete loss of *TNFAIP3*. In two of these cases (#6, and #41), sequence analysis had previously been performed [3], and only wild type sequence had been shown. Fourteen cases were assumed to have heterozygous deletions, of which nine had previously undergone sequence analysis revealing no mutations.

The remaining 25 cases were assumed to have no deletions, of which 14 had previous sequence data showing three cases (#3, #38, and #46) with gene mutations. The sequencing analysis revealed no residual wild-type *TNFAIP3*. In these cases, the relative TNFAIP3/CEP6 signal ratio was 0.72, 0.69, and 0.70, respectively.

We next carried out statistical analysis of the relationship between *TNFAIP3* deletions as detected by FICTION analysis and clinical data. The frequency of *TNFAIP3* deletions was higher in cases with B symptoms, although the difference was not statistically significant (*p* = 0.11) (Table 3). There were no significant differences related to the patient gender, cHL type, or the disease stage. Cases with mutated *TNFAIP3* were then combined with those with deletions, again revealing no statistically significant correlations with clinic pathological characteristics (data not shown).

**Table 3:** Univariate analysis

**Clinical characteristics**	**Number of alleles: 2**	**Number of alleles: 0,1**	***p*-value**
NS	14	12	0.63
Others	11	8	
I - II	17	12	0.74
III - IV	8	7	
B +	4	7	0.11
B -	21	12	
EBV +	8	6	0.98
EBV -	17	13	
M	10	7	0.83
F	15	12	

NS, Nodular sclerosis cHL; M, male; F, female.

The frequency of deletions was not significantly different between cases with and without evidence of EBV infection. However, all NS type cases with homozygous *TNFAIP3* deletions were found to be negative for EBV; among the remaining 40 cases, 13 tested positive for EBV.

## Discussion

FICTION analysis is useful to detect gain-of-function gene deletions in certain cells [4], and was used in the present study to evaluate the frequency of *TNFAIP3* deletions in cHL. In FISH analysis, use of thinner sections enables improved probe penetration and better signal visualization. However, as the average HR-S cell is 30–50 μm in diameter, this far exceeds the maximum FISH section thickness of 4 μm, so evaluation of signal number is difficult. We conducted the analysis in a large number of cells, calculated the TNFAIP3/CEP6 signal ratio and determined the cutoff level, which contributed to the determination of allele frequency at the *TNFAIP3* locus in cHL cases.

Of the 44 evaluable cases, 19 had deletions, which is consistent with the frequency reported in a previous deletion study (9/21) [7], confirming that *TNFAIP3* is frequently lost in malignant cells in cHL. Five cases had homozygous deletions and two of these had a normal sequence, possibly because of contamination from surrounding cells which cannot be avoided during microdissection. Thus, homozygous deletions might be missed in sequence analysis of HR-S cells. For this reason, our previous study only included cases in which the sequence analysis was successful. Cases with homozygous deletions might also have been excluded because of unsuccessful PCR amplification, irrespective of DNA quality. If these cases had been included, the frequency of *TNFAIP3* homozygous deletions might be much higher than previously thought.

The allele status of case #40 could not be determined because of the limited sample number; however, this case had a very low TNFAIP3/CEP6 signal ratio. A previous sequence analysis of the same case revealed both mutated and wild-type alleles. If it is assumed that the HR-S cells were diploid, the results could be sorted into homozygous, heterozygous, and no deletions. However, karyotype analysis previously demonstrated the cells to be triploid or tetraploid in some cases [11]. Therefore, it is possible that the mutated allele co-exists with two or three wild-type alleles in triploidy or tetraploidy, respectively. Alternatively, this could be explained by tumor cell heterogeneity in which HR-S is a mixture of cells with a mutated gene and cells with deleted genes.

Of the 14 cases identified to have heterozygous deletions, sequence analysis data were available for nine but no mutations were detected in any of these cases. This could be a result of the gene repression caused by promoter methylation, which was shown for MALT lymphoma by Chanudet et al. [7]. In cases of MALT and other lymphomas, *TNFAIP3* has been postulated to work as a tumor-suppressor gene. However, it has been difficult to determine allele loss in cHL cases because CGH analysis is laborious and requires large numbers of micro-dissected cells [12]. FICTION was used in the present study to overcome this limitation.

In the 25 cases thought to have no *TNFAIP3* loss, sequence data were available for 14 of which three showed *TNFAIP3* mutations and the chromatogram did not show the remaining wild-type allele. This result is compatible with that of previous allele-specific CGH analysis, which showed a significant number of B-cell lymphoma cases with uniparental disomy (UPD) [3]. Of the three cases with *TNFAIP3* mutations in the present study, loss of function was caused by UPD. However, for the remaining 11 cases in which both FICTION and sequence data were available, no *TNFAIP3* deletion was again noted.

We found that B symptoms were more frequent in cases with *TNFAIP3* deletions, although the difference was not statistically significant. Knockout experiments have suggested that *TNFAIP3* plays a role in the termination of inflammation [13]. As B symptoms represent the inflammatory process, we speculated that absence of *TNFAIP3* causes termination of the inflammatory process to fail, resulting in continuous activation of NF-κB signaling and classical Pel-Ebstein fever in cHL patients. However, to confirm this, further expression studies of the *TNFAIP3* gene product in more cases are required.

All five cases with *TNFAIP3* homozygous deletions in the present study were EBV-negative, as were four of the 14 cases of heterozygous deletion. Schmitz et al. previously found that the frequency of *TNFAIP3* mutations in EBV-negative cases was significantly higher than in EBV-positive cases [7]. As our study included fewer EBV-positive cases, we could not confirm this report and further study including methylation analysis of *TNFAIP3* will be necessary.

## Conclusion

Homozygous deletion of *TNFAIP3* in cHL cases might be more frequent than previously thought. Homozygous deletions were found in 11% of our cases, and our findings strongly suggest that *TNFAIP3* serves as a tumor-suppressor gene in cHL. Screening for *TNFAIP3* by FICTION analysis is a useful, cost-effective and rapid research tool to identify heterogeneity among cHL cases.

## Abbreviations

cHL: Classical Hodgkin lymphoma; CGH: Comparative genomic hybridization; FICTION: Fluorescence immunophenotyping and interphase cytogenetics as a tool for the investigation of neoplasm; FISH: Fluorescence in situ hybridization; HL: Hodgkin lymphoma; HR-S: Hodgkin Reed-Sternberg; LD: Lymphocyte-depletion; LR: Lymphocyte-rich; MC: Mixed cellularity; NS: Nodular sclerosis; UPD: Uniparental disomy.

## Competing interests

The authors declare that they have no competing interests.

## Authors’ contributions

JN, AMD, and HT performed the FICTION analysis. JN, NH, MK, KTa, MS, and HN carried out microdissection experiments. JN, MK, MS, KTa, and YK performed mutation analysis of TNFAIP3. FH prepared the FICTION probe. KTa, YA, WM, NS, DM, TW, YK, and KTo prepared tumor specimens. JN, SO and YK participated in study design, and YK and JN wrote the manuscript. All authors discussed the results and commented on the manuscript.

## Pre-publication history

The pre-publication history for this paper can be accessed here:

http://www.biomedcentral.com/1471-2407/12/457/prepub
